# Department of Error

**DOI:** 10.1016/S0140-6736(17)32702-2

**Published:** 2017-10-28

*Coleman RL, Oza AM, Lorusso D, et al, for the ARIEL3 investigators. Rucaparib maintenance treatment for recurrent ovarian carcinoma after response to platinum therapy (ARIEL3): a randomised, double-blind, placebo-controlled, phase 3 trial.* Lancet *2017; **390:** 1949–61*—In table 1 and figure 4 of this Article (published online first on Sept 12, 2017), the two categories for “Time to progression with penultimate platinum” should have been “6 to ≤12 months” and “>12 months”. In the last sentence of the second paragraph of the Results section, “rucaparib” was misspelt and the sentence should have read “Median progression-free survival in the intention-to-treat population was 10·8 months (8·3–11·4) in the rucaparib group versus 5·4 months in the placebo group (5·3–5·5; 0·36 [0·30–0·45]; p<0·0001).” Reference 20 should have read “National Cancer Institute. NCI Term Browser, CTCAE. April 29, 2010. https://nciterms.nci.nih.gov/ncitbrowser/pages/vocabulary.jsf?dictionary=CTCAE&version=4.03 (accessed Sept 7, 2016).” Figure S3B and figure S6A in the appendix have also been updated. These corrections have been made to the online version as of Oct 26, 2017, and the printed Article is correct.

